# Environmental and structural factors associated with bacterial diversity in household dust across the Arizona-Sonora border

**DOI:** 10.1038/s41598-024-63356-6

**Published:** 2024-06-04

**Authors:** Lauren D. Benton, Nicolas Lopez-Galvez, Chloe Herman, J. Gregory Caporaso, Emily K. Cope, Cecilia Rosales, Mercedes Gameros, Nathan Lothrop, Fernando D. Martínez, Anne L. Wright, Tara F. Carr, Paloma I. Beamer

**Affiliations:** 1 Department of Pediatrics, Steele Children’s Research Center, College of Medicine, University of Arizona Health Sciences, 1501 N. Campbell Avenue, Tucson, AZ 85724 USA; 2 Asthma and Airway Disease Research Center, University of Arizona, College of Medicine, University of Arizona Health Sciences, 1501 N. Campbell Avenue, Tucson, AZ 85724 USA; 3 Mel and Enid Zuckerman College of Public Health, University of Arizona, 1295 N. Martin Ave, PO 245210, Tucson, AZ 85724 USA; 4 San Diego State University Research Foundation, San Diego State University, 5250 Campanile Dr, San Diego, CA 92182 USA; 5 Center for Applied Microbiome Science, Pathogen and Microbiome Institute, Northern Arizona University, 1350 S Knoles Dr, Flagstaff, AZ 86011 USA; 6 Department of Biological Sciences, Northern Arizona University, Flagstaff, AZ USA; 7 School of Informatics, Computing and Cyber Systems, Northern Arizona University, Flagstaff, AZ USA

**Keywords:** Microbiology, Environmental sciences, Environmental social sciences

## Abstract

We previously reported that asthma prevalence was higher in the United States (US) compared to Mexico (MX) (25.8% vs. 8.4%). This investigation assessed differences in microbial dust composition in relation to demographic and housing characteristics on both sides of the US–MX Border. Forty homes were recruited in the US and MX. Home visits collected floor dust and documented occupants’ demographics, asthma prevalence, housing structure, and use characteristics. US households were more likely to have inhabitants who reported asthma when compared with MX households (30% vs. 5%) and had significantly different flooring types. The percentage of households on paved roads, with flushing toilets, with piped water and with air conditioning was higher in the US, while dust load was higher in MX. Significant differences exist between countries in the microbial composition of the floor dust. Dust from Mexican homes was enriched with *Alishewanella, Paracoccus*, *Rheinheimera* genera and *Intrasporangiaceae* family. A predictive metagenomics analysis identified 68 significantly differentially abundant functional pathways between US and MX. This study documented multiple structural, environmental, and demographic differences between homes in the US and MX that may contribute to significantly different microbial composition of dust observed in these two countries.

## Introduction

The indoor household environment, where people spend up to 90% of their time, contains a complex ecosystem of organisms, including trillions of microbes^1–3^. Some of these microorganisms adversely affect human health, while others can have a positive health impact^4–6^. Previous studies have demonstrated that indoor microbiome diversity can play an important role in the protection against immune-mediated diseases^7^. For example, there are microbial diversity differences in dust samples from houses of children without asthma versus children with asthma^8,9^. Urbanization has led to decreasing microbial diversity in people’s homes^10^. One proposed premise for the difference in microbiome diversity is that urbanization can result in more enclosed built environments with limited exposure to diverse microbiota^10–13^. Additionally, anthropogenic pollutants present in urban environments, along with high use of household disinfectants present in Western modern lifestyles, may alter the microbial balance in household environments by potentially reducing its biodiversity^14–16^. The microbial community in household environments can be linked to the number of occupants, both human and pets. Also, it has been proposed that house characteristics such as the design, humidity, type of ventilation, and the geographical location of the house could play an important role in shaping the microbial community^3,4,17^. Geography, however, may be a stronger driver of building environment microbiome composition than other factors such as building material^18^.

Regions that are similar in climate and population genetics but have different living conditions can illustrate the impact of varying household exposures on human health. For example, Finnish and Russian Karelia are two bordering regions that developed differently after World War II. Russian Karelia continued small scale farming and is, overall, a more rural environment, whereas Finnish Karelia went through rapid economic growth and urbanization. These areas also have different rates of atopic disease, proposed to be related to differences in microbial exposures^19–21^..Analogous environmental differences can be observed between East and West Germany, as well as between Amish and Hutterite communities^22,23^. After the Berlin Wall fell, East and West Germany were very different, with West Germany being more modernized and wealthier; this led to West Germany having higher rates of allergy and asthma^23,24^. Children who grow up on traditional European farms have less prevalence of asthma, and this effect may be in part due to exposure to a greater diversity of microbes in early life^22,25,26^. The prevalence of asthma is much greater in Hutterite communities compared to Amish communities even though they share similar genetic ancestry. This is thought to be due to differences in farming practices, as the populations have similar genetic ancestry. The Amish communities practice more traditional farming, whereas the Hutterite use modern farming techniques^22^. Together, we see that evaluating areas with similar geographic, climatic, and genetic background of inhabitants allows us to examine how socioeconomic, land use, and environmental determinants contribute to health disparities, such as the development of chronic diseases like asthma.

Another striking example of political and economic divide impacting similar ethnic and geographic areas can be observed along the US–Mexico Border. Nogales, Arizona, US, and Nogales, Sonora, MX are sister cities along the US–Mexico Border, that together are called Ambos Nogales (i.e., Both Nogales). These cities were once united with shared cultures, families, and economies, but due to changing political pressures, became divided, similar to East and West Berlin. Nogales, MX is the larger of the two cities, with a population of 220,292 in 2010, compared to the 20,837 residents in Nogales, US. Tucson, Arizona, US is located only about 100 km north of Ambos Nogales, and although it has a much larger population (close to 1 million) it is much less densely populated (U.S. Census Bureau 2012; Gobierno Municipal Nogales Sonora 2010). There are measurable infrastructure differences between the US cities and Nogales, MX, as the cities located in the US have similar access to the US standards of sanitation, clean drinking water, and the American style of housing development. Within Nogales, MX, there are distinct neighborhoods with differing socioeconomic status (SES), which leads to different housing styles and variable access to municipal infrastructure. Some neighborhoods are similar to US neighborhoods with piped water and planned housing developments. Others, the *colonia marginadas,* or informal settlements, do not have access to municipal resources such as sanitation and drinking water^27,28^.

We previously found that there is four-fold higher prevalence of asthma amongst children of Mexican descent attending middle school in southern Tucson and Nogales, US compared to those attending middle school in Nogales, Sonora, MX, despite similar ethnic backgrounds and a limited geographic region^29^. We found the lowest rates of asthma at the school with lower socioeconomic status located in a *colonia marginada*. These findings lead to questions about whether there are differences in household microbial environments in the different cities. The objective of this study was to identify differences in housing characteristics, dust load, and dust microbial diversity in households located on both sides of the US–MX Border in Tucson, Arizona, US (TUS), Nogales, Arizona, US (NUS), and Nogales, Sonora, MX (NMX) that may contribute to asthma prevalence. This study also investigated two different neighborhoods within Nogales, MX, including a traditionally high SES neighborhood and low SES neighborhood for differences in housing characteristics, dust load, and dust microbial diversity.

## Results

### Study population

Overall, 20 homes from Tucson, Arizona, US (TUS), 20 homes from Nogales, Arizona, US (NUS), and 40 homes from Nogales, Sonora, MX (NMX) (20 high socioeconomic status (high SES) and 20 low socioeconomic status (low SES) were recruited into the study. The household and environmental characteristics were analyzed and compared between the countries (US vs MX) as well as between neighborhoods (TUS, NUS, high SES NMX, and low SES NMX). There was no significant difference in age (mean years of age ± SD US = 29 ± 6, MX = 32 ± 11 *p* = *0.58*) or gender (female gender: US = 93.0%, MX = 90%, *p* = 0.50) of the respondents between the US and MX, but the respondents in the US had significantly more years of education (mean years of education ± SD US = 14 ± 4, MX = 11 ± 4, *p* < 0.01) (Supplemental Table S1). US households had higher incomes (*p* < 0.01) and cleaned their floors less frequently (*p* < 0.01) (Table 1). The source of drinking water was also significantly different between homes in MX and homes in the US, with more US homes utilizing public tap water (*p* < 0.01) (Table 1). US families were significantly more likely to have inhabitants with asthma than those in MX (US = 30.0%, MX = 5.0%, *p* < 0.01) (Table 1). There were no significant differences in homes that had smokers (US = 29%, MX = 28%, *p* = 1.00, or number of inhabitants per household (US = 6.2 ± 2.2, MX = 6.4 ± 2.0, *p* = 0.52) between the US and MX (Table 1). There was a significant difference in whether the households had any pets between US and MX (*p* < 0.01) as well as between TUS vs NUS vs high SES NMX vs low NMX (*p* = 0.01) (Table 1).

**Table 1:** Characteristics of the occupants and households.

Variable	L SES NMX n(%)	H SES NMX n(%)	MX n(%)	NUS n(%)	TUS n(%)	US n(%)	*p*-value for cities (Fisher’s Exact)	*p*-value for US–MX (Fisher’s exact)
Someone with asthma lives in the home	1 (5.0)	1 (5.0)	**2 (5.0)**	6 (30.0)	6 (30.0)	**12 (30.0)**	0.04	** < 0.01**
Household Income								
< $6,000								
$6,000-$9,999	20 (100)	14 (70.0)	**34 (85.0)**	0 (0.0)	0 (0.0)	**0 (0.0)**	< 0.01	** < 0.01**
$10,000-$15,999	0 (0.0)	5 (25.0)	**5 (12.5)**	0 (0.0)	1 (5.0)	**1 (2.5)**		
$16,000-$20,999	0 (0.0)	1 (5.0)	**1 (2.5)**	2 (10.0)	8 (40.0)	**10 (25.0)**		
$21,000-$25,999	0 (0.0)	0 (0.0)	**0 (0.0)**	7 (35.0)	3 (15.0)	**10 (25.0)**		
≥ $26,000	0 (0.0)	0 (0.0)	**0 (0.0)**	3 (15.0)	3 (15.0)	**6 (15.0)**		
	0 (0.0)	0 (0.0)	**0 (0.0)**	8 (40.0)	5 (25.0)	**13 (32.5)**		
Smokers live in the home	15 (75.0)	13 (65.0)	**28 (70.0)**	14 (70.0)	15 (75.0)	**29 (72.5)**	0.95	**1.00**
Homes with at least 1 animal	14 (70.0)	5 (25.0)	**19 (47.5)**	6 (30.0)	5 (25.0)	**11 (27.5)**	0.01	** < 0.01**
Frequency of floor cleaning*								
Several times a day	1 (5.0)	8 (40.0)	**9 (23.1)**	0 (0.0)	2 (10.0)	**2 (5.0)**	< 0.01	** < 0.01**
Everyday	16 (84.0)	8 (40.0)	**24 (87.2)**	3 (15.0)	7 (35.0)	**10 (25.0)**		
Several times a week	0 (0.0)	4 (20.0)	**4 (10.3)**	14 (70.0)	10 (50.0)	**24 (60.0)**		
Once a week	2 (11.0)	0 (0.0)	**2 (5.1)**	2 (10.0)	0 (0.0)	**2 (5.0)**		
< Once a month	0 (0.0)	0 (0.0)	**0 (0.0)**	1 (5.0)	1 (5.0)	**2 (5.0)**		
Primary Drinking Water Source**								
Indoor, public	1 (5.0)	0 (0.0)	**1 (2.6)**	4 (22.0)	6 (30).0	**10 (26.3)**	< 0.01	** < 0.01**
Bottled	3 (15.0)	16 (84.0)	**19 (48.7)**	13 (72.0)	8 (40.0)	**21 (55.3)**		
Hauled in	16 (80.0)	1 (5.0)	**17 (43.6)**	0 (0.0)	0 (0.0)	**0 (0.0)**		
Vending machine	0 (0.0)	2 (11.0)	**2 (5.1)**	1 (6.0)	6 (30.0)	**7 (18.4)**		
Number of people in household								
≤ 3	3 (15.0)	3 (15.0)	**6 (20.0)**	7 (35.0)	3 (15.0)	**10 (10.0)**	0.05	**0.07**
4	4 (20.0)	8 (40.0)	**12 (30.0)**	6 (30.0)	6 (30.0)	**12 (30.0)**		
5	2 (10.0)	4 (20.0)	**6 (15.0)**	4 (20.0)	6 (30.0)	**10 (25.0)**		
6	4 (20.0)	5 (25.0)	**9 (22.5)**	0 (0.0)	1 (5.0)	**1 (2.5)**		
≥ 7	7 (35.0)	0 (0.0)	**7 (17.5)**	3 (15.0)	4 (20.0)	**7 (17.5)**		

First *p*-values column are *p-values* comparing the low socioeconomic neighborhood of Nogales, Mexico vs the high socioeconomic neighborhood of Nogales, Mexico vs Tucson, United States of America vs Nogales, United States of America (value for cities). Second bold *p*-values column are comparing homes in Mexico vs in the United States of America Abbreviations: LSES NMX = low socioeconomic status Nogales, Sanora, Mexico; HSES NMX = high socioeconomic status Nogales, Sanora, Mexico; MX = Mexico; NUS = Nogales, Arizona, United States of America; TUS = Tucson, Arizona, United States of America; US = United States of America *1 of the Mexico homes did not have info on frequency of floor cleaning. **1 of the Mexico homes and 2 of the Arizona homes did not have info on water source.

### Household characteristics

Although all cities were close to the US–MX Border and within 100 km of each other, there were striking differences between the homes by country. All the homes in high SES NMX had the same floor plan, as it was a planned subdivision. The percentage of homes with paved roads (*p* < 0.01), flushing toilets (*p* < 0.01), and piped water (*p* < 0.01)was significantly lower in low SES NMX (Table 2) compared with the other neighborhoods. The homes in the US differed from those in MX in type of structure (Table 2), with 33% of homes in US being apartments or trailers, while 100% of the homes in MX were detached or duplex houses (*p* < 0.01) (Table 2). Homes in the US had more rooms and more bathrooms than in MX (*p* < 0.01). All homes in the US had either air conditioning, evaporative cooling, or both, whereas only a few of the homes in MX (10%) had either type of cooling (*p* < 0.01) (Table 2). Flooring also differed between the US and MX. Most of the rooms where the dust samples were collected in MX had smooth floors (93%), whereas 42% of samples in the US were collected from carpet or rugs (*p* < 0.01) (Table 2). Significantly more homes in TUS and low SES NMX had mildew or moisture present than the other sites (TUS = 25.0%, NUS = 0.0%, high SES NMX 0.0%, low SES NMX 5.0%, *p* = 0.01) (Table 2).

**Table 2:** Physical characteristics of the homes in Mexico vs in the United States of America, and the low socioeconomic neighborhood of Nogales, Mexico vs the high socioeconomic neighborhood of Nogales, Mexico vs Tucson, United States of America vs Nogales, United States of America.

Variable	LSES NMX n(%)	HSES NMX n(%)	MX n(%)	NUS n(%)	TUS n(%)	US n(%)	*p*-value for cities (Fisher Exact)	*p*-value for US–MX (Fisher Exact)
Piped Water								
No	18 (90.0)	0 (0.0)	**18 (45.0)**	0 (0.0)	0 (0.0)	**0 (0.0)**	< 0.01	** < 0.01**
Yes	2 (10.0)	20 (100.0)	**22 (55.0)**	20 (100.0)	20 (100.0)	**40 (100.0)**		
Flushing toilets								
No	16 (80.0)	0 (0.0)	**16 (40.0)**	0 (0.0)	0 (0.0)	**0 (0.0)**	< 0.01	** < 0.01**
Yes	4 (20.0)	20 (100.0)	**24 (60.0)**	20 (100.0)	20 (100.0)	**40 (100.0)**		
Road Type								
Unpaved, dirt	20 (100.0)	0 (0.0)	**20 (50.0)**	0 (0.0)	1 (5.0)	**1 (2.5)**	< 0.01	** < 0.01**
Asphalt	0 (0.0)	20 (100.0)	**20 (50.0)**	20 (100.0)	19 (95.0)	**39 (97.5)**		
Type of Structure ~								
Detached	19 (100.0)	8 (40.0)	**27 (69.2)**	10 (50.0)	14 (70.0)	**24 (60.0)**	< 0.01	** < 0.01**
Duplex/attached	0 (0.0)	12 (60.0)	**12 (30.8)**	3 (15.0)	0 (0.0)	**3 (7.5)**		
Multi-unit (apartment)	0 (0.0)	0 (0.0)	**0 (0.0)**	7 (35.0)	2 (10.0)	**9 (22.5)**		
Trailer/mobile	0 (0.0)	0 (0.0)	**0 (0.0)**	0 (0.0)	4 (20.0)	**4 (10.0)**		
Number of rooms*								
1	5 (25.0)	0 (0.0)	**5 (12.5)**	0 (0.0)	2 (10.0)	**2 (5.0)**	< 0.01	** < 0.01**
2**	6 (30.0)	20 (100.0)	**26 (65.0)**	4 (20.0)	1 (5.0)	**5 (12.5)**		
3	4 (20.0)	0 (0.0)	**4 (10.0)**	10 (50.0)	2 (10.0)	**12 (30.0)**		
4	4 (20.0)	0 (0.0)	**4 (10.0)**	4 (20.0)	10 (50.0)	**14 (35.0)**		
5	1 (5.0)	0 (0.0)	**1 (2.5)**	2 (10.0)	4 (20.0)	**6 (15.0)**		
6	0 (0.0)	0 (0.0)	**0 (0.0)**	0 (0.0)	1 (5.0)	**1 (2.5)**		
Number of bathrooms								
0	1 (5.0)	0 (0.0)	**1 (2.5)**	0 (0.0)	0 (0.0)	**0 (0.0)**	< 0.01	** < 0.01**
1**	18 (90.0)	20 (100.0)	**38 (95.0)**	5 (25.0)	7 (35.0)	**12 (30.0)**		
1.5	0 (0.0)	0 (0.0)	**0 (0.0)**	1 (5.0)	0 (0.0)	**1 (2.5)**		
2	1 (5.0)	0 (0.0)	**1 (2.5)**	12 (60.0)	12 (60.0)	**24 (60.0)**		
3	0 (0.0)	0 (0.0)	**0 (0.0)**	2 10.0)	1 (5.0)	**3 (7.5)**		
Cooling System								
Air conditioning	2 (10.0)	1 (5.0)	**3 (7.5)**	14 (70.0)	9 (45.0)	**23 (57.5)**	< 0.01	** < 0.01**
Evaporative cooler	1 (5.0)	0 (0.0)	**1 (2.5)**	6 (30.0)	6 (30.0)	**12 (30.0)**		
Both	0 (0.0)	0 (0.0)	**0 (0.0)**	0 (0.0)	5 (25.0)	**5 (12.5)**		
None	17 (85.0)	19 (95.0)	**36 (90.0)**	0 (0.0)	0 (0.0)	**0 (0.0)**		
Type of floor								
All carpet/rug	0 (0.0)	0 (0.0)	**0 (0.0)**	9 (45.0)	8 (40.0)	**17 (42.5)**	< 0.01	** < 0.01**
Both/mixture	1 (5.0)	1 (5.0)	**2 (5.0)**	1 (5.0)	2 (10.0)	**3 (7.5)**		
All smooth floor	18 (95.0)	19 (95.0)	**37 (92.5)**	10 (50.0)	10 (50.0)	**20 (50.0)**		
Dirt floor	1 (5.0)	0 (0.0)	**1 (2.5)**	0 (0.0)	0 (0.0)	**0 (0.0)**		
Evidence of moisture or mildew								
No	19 (95.0)	20 (100.0)	**39 (97.5)**	20 (100.0)	15 (75.0)	**35 (87.5)**	0.01	**0.20**
Yes	1 (5.0)	0 (0.0)	**1 (2.5)**	0 (0.0)	5 (25.0)	**5 (12.5)**		

First *p*-values column are *p-values* comparing the low socioeconomic neighborhood of Nogales, Mexico vs the high socioeconomic neighborhood of Nogales, Mexico vs Tucson, United States of America vs Nogales, United States of America (value for cities). Second bold *p*-values column are comparing homes in Mexico vs in the United States of America.. Statistical significance was determined using Fisher’s Exact Test. Abbreviations: LSES NMX = low socioeconomic status Nogales, Sanora, Mexico; HSES NMX = high socioeconomic status Nogales, Sanora, Mexico; MX = Mexico; NUS = Nogales, Arizona, United States of America; TUS = Tucson, Arizona, United States of America; US = United States of America ~ 1 home structure in LSES NMX had no info on structure *excludes bathrooms; ** confirmed, all houses in HSES neighborhood in NMX had the same floor plan in a subdivision.

### In-home floor dust loading

As presented in Table 3, we found significantly greater amounts of dust loading in MX homes (US = 107.4 ± 2.2 mg/m^2^ MX = 172.4 ± 2.9 mg/m^2^, *p* = 0.04) compared to US homes. In addition, the homes located on a dirt road had significantly higher dust load than the homes located on an asphalt road (dirt = 285.9 ± 3.0 mg/m^2^ asphalt/tile = 106.3 ± 2.2 mg/m^2^, *p* = 0.02). Significantly more dust was retrieved in homes with at least one pet (homes with a pet = 176.9 ± 3.27 mg/m^2^, homes with no pet 115.3 ± 2.15 mg/m^2^, *p* = 0.01), more than two children ( more than 2 children = 192.1 ± 3.2 mg/m^2^, 2 or less than 2 children = 115.3 ± 2.3 mg/m^2^, *p* = 0.01), and more than 4 residents ( more than 4 resident = 176.4 ± 2.9 mg/m^2^, 4 or less adults = 105.1 ± 2.2, *p* = 0.01) (Table 3). There were no significant differences in dust loading based on the number of rooms or number of adults living in the home. Finally, although we did not find any significant differences between the dust load by floor type in homes located in the US versus MX, there was a significantly higher (*p* = 0.03) dust load within US homes that had some sort of carpet, in comparison to homes with smooth flooring (Supplemental Fig. S1). There were no differences in dust loading in relation to whether there were asthmatic inhabitants (*p* = *0.89)*.

**Table 3:** Floor dust loading (mg/m^2^) in relation to household characteristics. Statistical significance was determined using Mann Whitney U Test.

Variable	N	Geomean	GSD	*p*-value
Location				
US	40	107.4	2.19	0.04
MX	40	172.4	2.93	
Self-Reported asthma				
No one with asthma	66	137.7	2.79	0.89
At least one person with asthma	14	128.9	1.89	
Nearest road type				
Dirt	21	285.9	3.03	0.02
Asphalt/Tile	59	106.3	2.18	
Number of rooms				
2 or less bedroom	38	166.7	2.76	0.04
More than 2 bedrooms	42	113.3	2.44	
Pets				
No pets	50	115.3	2.15	0.01
Owning at least one pet	30	176.9	3.27	
Number of adults				
2 or less adults	47	130.9	2.11	0.87
More than 2 adults	33	143.8	3.40	
Number of children				
2 or less children	54	115.3	2.27	0.01
More than 2 children	26	192.1	3.19	
Total number of residents				
4 or less residents	40	105.1	2.16	0.01
More than 4 residents	40	176.4	2.94	

MX = Mexico; US = United States of America.

### Dust microbiome

**Figure 1:**
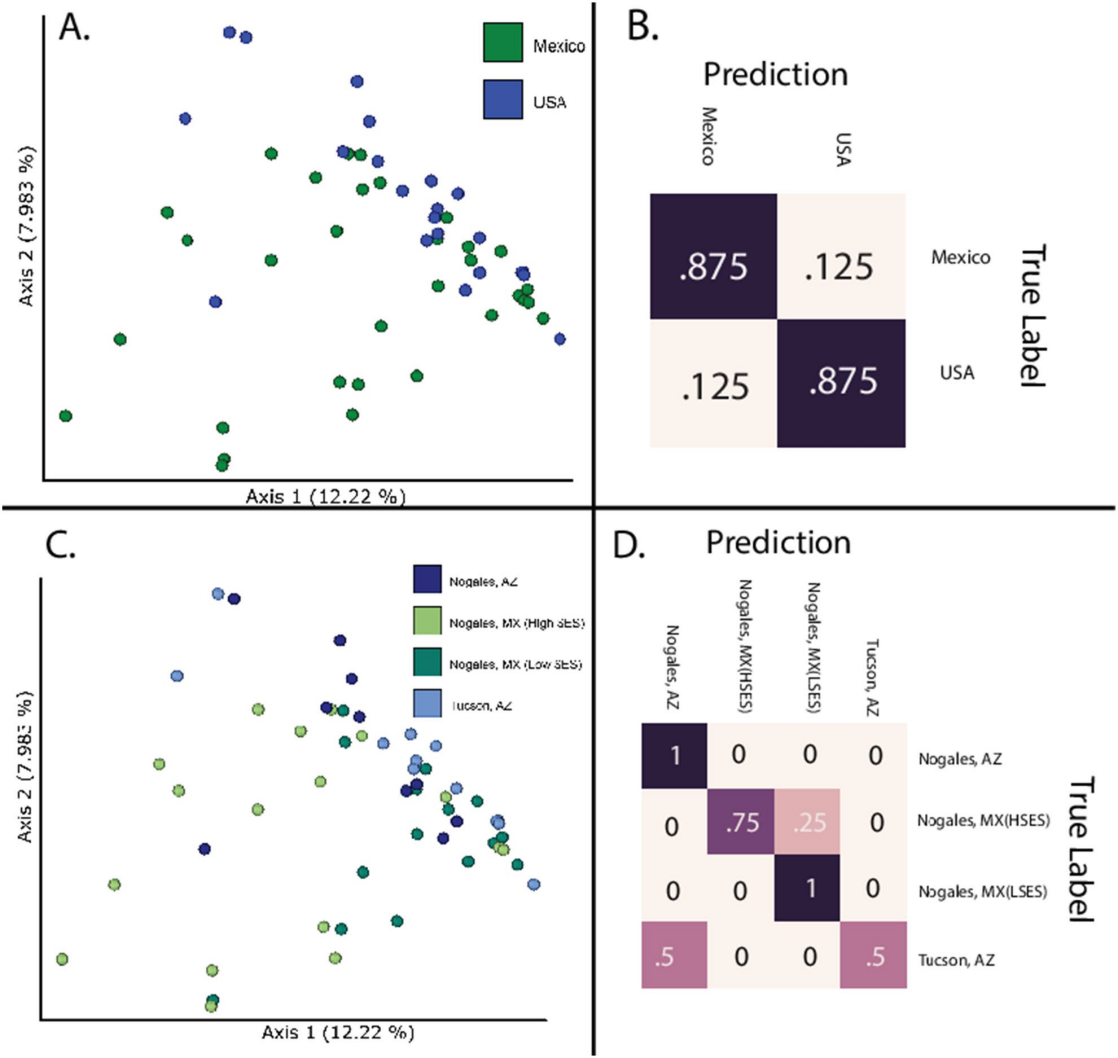
(**A**) Unweighted UniFrac beta diversity plot comparing the United States of America (US) and Mexico (MX) house dust (PERMANOVA pseudo-F = 3.02 *p* < 0.001, q < 0.0015). (**B**) Random Forest classifier trained to differentiate the neighborhoods using the dust microbiome compositions. The classifier achieved an overall accuracy of 0.87594. US samples were accurately labeled 87.5100% of the time, and Mexican samples were accurately labeled 89% of the time. (**C**) Unweighted UniFrac beta diversity plot comparing high SES NMX neighborhood vs low SES NMX, vs NUS vs TUS. (**D**) Random Forest classifier trained to differentiate the neighborhoods using the dust microbiome compositions the model predicted NUS accurately 10,075% of the time; TUS 560% of the time: high SES NMX 75% of the time; and low SES NMX 100% of the time. LSES = low socioeconomic status Nogales, Sanora, Mexico; HSES = high socioeconomic status Nogales, Sanora, Mexico; MX = Mexico; USA = United States of America.

**Figure 2:**
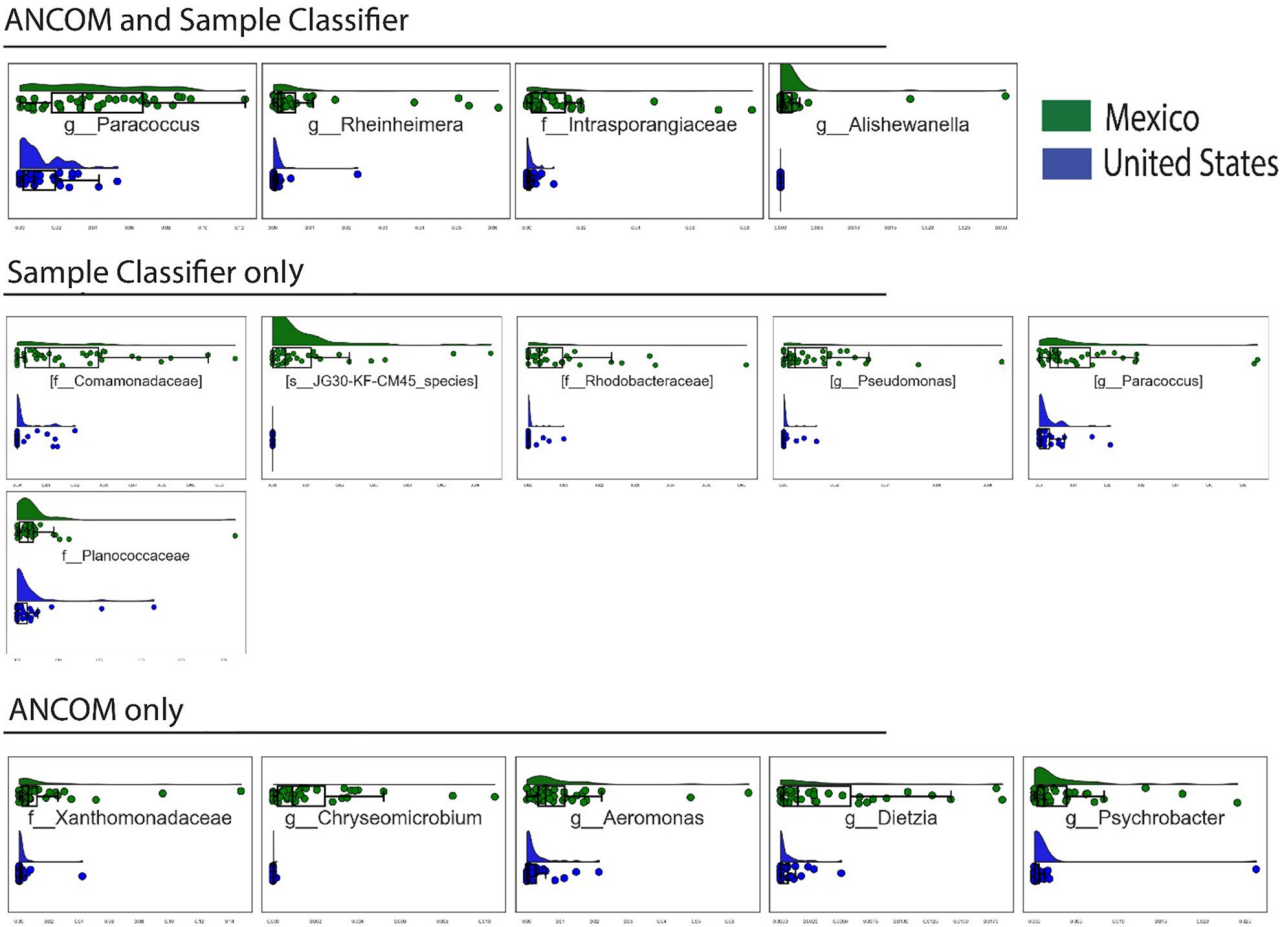
ANCOM and Sample Classifier comparing the house dust from homes in the United States versus Mexico. Top is Mexico and bottom is United States of America. The x-axes in these figures represent the relative abundance of the taxon that is highlighted in the panel, while the y-axes represent the categorizations of the samples. Each point therefore represents the relative abundance of a taxon in a single sample, and the box plots and the histograms show the distribution of the relative abundances in each sample group.

The microbiome compositions, as measured by unweighted UniFrac, of the dust in the US samples and MX samples were significantly different (PERMANOVA pseudo-F = 2.59 *p* < 0.001, q < 0.001; Fig. 1). We trained a random forest classifier to differentiate the neighborhoods using the dust microbiome compositions, and the classifier achieved an overall accuracy of 0.875. A standard approach for assessing accuracy is to compare this accuracy to the accuracy that would be achieved with a “dummy classifier”. Typically, this would be “classifying” based on randomly assigning a label to each sample, or assigning the most frequent label to each sample, and computing the resulting accuracy. When we compare the accuracy of our random forest classifier to baseline accuracy achieved with a “most frequent label” dummy classifier, we found that the random forest classifier’s accuracy was 1.75 times better than baseline accuracy (Fig. 1B). This supports our PERMANOVA results by suggesting that our dust microbiome feature data contains information that is specific to the different neighborhoods. Samples predicted to be from the United States were accurately labeled 87.50% of the time, and samples from Mexico were accurately labeled 87.5% of the time (Fig. 1B). Our random forest model and ANCOM each reported many different genera, but both methods identified three of the same genera and one of the same family that were enriched in the MX dust (*Alishewanella*, *Paracoccus*, *Rheinheimera* genera and *Intrasporangiaceae* family) (Fig. 2).

The dust microbial composition between the four sites (two US cities and two MX neighborhoods) was significantly different (PERMANOVA pseudo-*F* = 2.06, *p* < 0.001; because all tests’ results reported in this paragraph were the same, we will report the results as *F*, *p*, and *q*-values for readability). The microbial composition in the dust from the higher SES neighborhood in NMX was significantly different from the US cities’ (NUS versus high SES NMX): *F* = 1.74, *p* = 0.011, *q* = 0.0132; TUS versus high SES NMX): F = 2.50, *p* = 0.003, q = 0.0030) and significantly different from low SES NMX: (*F* = 2.35, *p* = *0.003*, *q* = 0.0045). The low SES NMX neighborhood dust microbiome composition was also significantly different compared to the US cites (NUS versus low SES NMX): *F* = 2.18, *p* < 0.001, *q* = 0.003; TUS versus low SES NMX: *F* = 2.227, *p* < 0.001, *q* = 0.0030) (Fig. 1). The US cities were not significantly different from each other (TUS versus NUS: *F* = 1.20, *p* = 0.133, *q* = 0.133 (Fig. 1). A sub-analysis showed that the house dust microbial composition differed when comparing the homes by road type (*F* = 2.21, *p* < 0.001, *q* < 0.001), presence of air conditioning (air conditioning vs none: *F* = 1.86, *p* = 0.006, *q* = 0.036), all rug vs all smooth flooring present in the home (*F* = 1.79, *p* = 0.009*, q* = 0.027, having household income of < $6,000 (the lowest bracket of income) vs ≥ $26,000 (the highest bracket of income) (*F* = 1.87, *p* = 0.003*, q* = 0.045, having piped water at the house (*F* = 1.75, *p* = 0.008), having flushing toilets in the home (*F* = 1.52, *p* = 0.031), and main drinking water source for the house (hauled in vs indoor public water (*F* = 1.56 *p* = 0.005*, q* = 0.015) and hauled in vs vending machine water (*F* = 1.62, *p* = 0.005*, q* = 0.015)).(Supplemental Fig. S2 and Supplemental Fig. S2 Supplemental table S1). This also showed the house dust microbial composition was significantly different in houses that had an asthmatic vs those that did not have an asthmatic present in the home (*F* = 1.14, *p* = 0.018*, q* = 0.019) (Supplemental Fig. S2)).We trained a random forest classifier to differentiate the neighborhoods using the dust microbiome compositions, and the classifier achieved an overall accuracy of 0.81, which was 3.25 times better than baseline accuracy (assigning the most common category to all samples). The model’s NUS predictions were accurate 100% of the time; the TUS predictions were accurate 50% of the time, where the remaining 50% of the TUS samples were labeled NUS; the high SES NMX predictions were all correct 75% of the time, where the remaining 25% of the time the high SES NMX samples were predicted to be from low SES NMX; and the low SES NMX samples were all accurately classified (Fig. 1). When the model predicted the wrong neighborhood, it predicted the correct country. Our random forest model and ANCOM for comparing neighborhoods both found one genera that were enriched in the dust microbiome the low SES NMX group (*Georgenia)* (Supplemental Fig. S3).

The PICRUSt analysis predicted that there were 68 significantly differentially abundant functional pathways in the house dust microbial communities present when comparing the US to MX (Supplemental Fig. S4). Twenty-two significantly different functional pathways of the microbial communities in the dust existed between high SES NMX vs low SES NMX vs NUS, as compared to TUS (Supplemental Fig. S5). The PICRUSt analysis predicts that the microbes present in the house dust in low SES NMX had superpathway of polyamine biosynthesis III capabilities. When comparing the low SES NMX to the high SES NMX, the microbes present in the house dust in the low SES NMX neighborhood had greater pyrimidine deoxyribonucleotides biosynthesis, benzoyl CoA anaerobic degradation, nitrate reduction, and spirilloxanthin and 2.2-diketo-spirilloxanthin biosynthetic capabilities (Supplemental Fig. S5).

## Discussion

This study examined differences in household characteristics, environmental factors, socio-economic factors, and microbial composition of dust from households in neighborhoods across the US–MX Border, in which we previously documented differential rates of asthma prevalence^29^. We identified multiple structural, environmental, and human factor differences between the US and MX homes. There were differences in dust loading and microbial diversity across the samples collected from the homes. Some of the key structural differences between the homes in MX vs the US may have led to the differences in microbial compositions. The diversity of microbial composition was significantly different when comparing household dust from homes with different road types, different drinking water sources, presence of air conditioning vs no air conditioning, all rug vs all smooth flooring, piped water at the house, and flushing toilets in the home. The bacterial genera *Alishewanella*, *Paracoccus*, *Rheinheimera* and the *Intrasporangiaceae* family were found to be enriched in the dust from homes within MX. In low SES NMX (low socioeconomic Nogales, Mexico) group the genus was *Georgenia* was found to be enriched in the house dust.

This study found multiple housing characteristics that differed between MX and the US, differences that may contribute to the higher prevalence of asthma in children of Mexican descent living in the US compared to MX^29^. Homes in the US were more likely to be on paved roads and to have flushing toilets, piped/municipal water, more rooms and bathrooms, and evaporative cooling and/or air conditioning compared to MX homes. Although only 26.3% of individuals in the US drank their tap water, this was significantly higher than in MX (2.6%). Floors were more likely to be carpeted in the rooms where the dust was collected in the US. Carpets in the home may be an important risk for asthma development as they can be a reservoir for mildew, mold, allergens, and chemical hazards (e.g., pesticides, metals, flame retardants, per-and polyfluoroalkyl substances (PFAS), and can contribute to poor indoor air quality^30,31^. Although carpets typically have greater dust loading than hard floors, the homes in MX had significantly greater dust loading than the ones in the US, despite the lack of carpet (Tables 2, 3). This suggests that differences in the household density (particularly the number of children), the presence of pets, and other household structural factors between these communities might account for the greater dust loading in MX.

Microbial populations in indoor environments, where we live and eat, play an important role in human health. Environmental dust exposure early in life appears to influence what bacteria colonize the gut, skin, and nasal microbiome^32–35^. It has been proposed that part of the reason for increasing asthma and allergy prevalence worldwide is due to shifts in our lifestyles towards more Western or Modernized ways of living, which has led to a decrease in microbial exposure during a critical period of immune development^36^. Individuals with exposure to more diverse bacteria have lower rates of allergic diseases^37,38^. We found significant differences in the microbiome composition of dust collected from homes in MX compared to the US (Fig. 1). Previous studies have shown a link between house dust microbial composition and risk of allergic asthma development. Children raised in Amish communities have lower rates of allergic sensitization and asthma than those from Hutterite communities. Stein et al. demonstrated that house dust from an Amish community had a different microbial composition than the house dust from a Hutterite community. Further, mice that received the dust from the Hutterite houses intranasally had decreased airway reactivity and eosinophilia, which are markers of allergic asthma^22^. In this study we found a significant difference in microbial dust composition between homes with and without an occupant that reports having asthma (Supplemental Fig. S2).

House dust microbial composition is affected by outdoor and indoor environments, including structural characteristics, as well as household occupants and their activities in the home. Any combination of the significant differences between the homes in MX and the US may have led to the differences observed in the microbial composition of the house dust. A sub-analysis showed that there was a difference in beta diversity (unweighted UniFrac) of the dust microbiome when comparing the homes by road type, presence of air conditioning, presence of piped/municipal water, presence of flushing toilets, drinking water sources, and having all rug vs all smooth flooring. Using an air conditioner changes the indoor environment by changing the temperature and humidity, which would lead to differences in microbial growth indoors^39^, as well as through filtering of the air. Homes with air conditioners are less likely to have windows open, and therefore less dust is likely to blow into the home. Furthermore, it has been shown that as regions become more industrialized and homes are constructed in a manner where they are more tightly sealed, the microbiome diversity decreases^40^. The water sources present in the homes caused variation in the house dust microbial composition. It has been shown that municipal water has fewer commensal microbes present and that drinking of municipal water is associated with higher prevalence of asthma and allergies^21,41^.

We have previously shown that MX and the US have different prevalence of childhood asthma, and this study also found differences in house dust composition between the two regions^29^. The dust from Mexican homes was more enriched with *A Alishewanella*, *Paracoccus*, *Rheinheimera* genera and *Intrasporangiaceae* family. Although *Alishewanella*, *Rheinheimera* genera and *Intrasporangiaceae* family have not been previously identified as related to asthma prevalence they have been linked to less industrialized and outdoor environments as well as other allergic diseases. *Alishewanella, Rheinheimera* and *Pararcoccus* are gram-negative bacteria that have endotoxin present in their cell walls. Previous studies have shown that higher levels of endotoxin in a child's environment are related to lower incidence of atopic asthma and allergic diseases^22,42,43^. *Alishewanella, Pararcoccus*, *Intrasporangiaceae,* and *Rheinheimera* and can be found in soil. *Intrasporangiaceae* has specifically been found to be in higher concentrations in soil in unindustrialized areas^44,45^. In the southwest of the US and in Mexico City, *Rheinheimera* has been found to be more frequently present in the air during dust storms and in the air of semirural area compared to urban areas^46,47^. Given that the household in MX where more likely to have dirt roads, no air-cooling system, and have greater air exchange rates with outdoors, it would be reasonable that these bacteria are more commonly present in MX homes. *Intrasporangiaceae* has been shown to be depleted in household dust in more industrialized regions^45,48^. Depletion of *Intrasporangiaceae* and *Rheinheimera* in a person’s environment and/or on their skin is linked to development of atopic dermatitis^49,50^*. Pararcoccus* has postulated to be protective against atopic dermatitis in that it is more prevalent in the skin microbiome of healthy adults to adults with skin affected by atopic dermatitis^51^. However, unlike all the rest of the identified genera or family it has previously been linked to asthma prevalence. In a study examining the effects of indoor microorganisms on asthma and allergic disease in children it was found that healthy children had house dust enriched with *Pararcoccus*^52^. *Alishewanella* was only recently established as a unique genus in the year 2000 and its diversity is still being understood. *Alishewanella* is also in the phylum of Proteobacteria*,* which, much like Actinobacteria*,* has been shown to be both negatively and positively associated with allergic disease in different studies^53–56^. The inconsistencies in the direction of these relationships may be explained by the need to classify beyond the phylum level, which is very broad, or the presence of critical time points at which exposure protects against onset of asthma but may be harmful once asthma has developed. In any case, the differences found in microbial exposure between the sites suggest promising areas for future research related to asthma in the Border region.

Although some microbes in the dust are unlikely to be active metabolically, others are living and metabolically active, and so may play a critical role in alteration of host microbiome. They can colonize the host, where they can become metabolically active and play a key role in risk of asthma development. A PICRUSt analysis was done to identify potentially relevant metabolic pathways present in the house dust from homes located in MX vs US. There were 23 biosynthetic pathways, 38 degradation/utilization/assimilation pathways, and 7 pathways involved in generation of precursor metabolites that were higher in the house dust from MX. Some of the pathways found to be more prevalent in the house dust from MX are involved in biosynthesis of molecules that are known to be protective against asthma (Supplemental Fig. S4), such as short chain fatty acids and pyrimidine^57,58^. There have been multiple studies that show the importance of metabolites produced by the microbiota that colonize humans in protecting against asthma via alterations in the epithelial barrier function and immune system regulations. Many of the possibly up-regulated functional pathways present in the house dust from MX have been examined in other studies and have been shown to be protective against asthma or helpful in asthma control. For example, the UDP − N − acetyl − D − glucosamine and the mycolate biosynthetic pathways that were higher in the house dust from MX have been shown to down-regulate allergic airway inflammation^58–62^ (Supplemental Fig. S4).

Limitations of this study include that collection of the house dust microbiome samples occurred at a single time-point and during a single season. There was significant differences in the ambient temperatures during sample collection across the border and this was likely related to more of the MX samples being collected in the spring/summer months and more of the US sample being collected in the winter. Future studies would benefit by looking at multiple time points to assess the variability of household microbiomes. The high SES NMX homes were all part of a subdivision with identical floor plans, which could drive some of the differences observed between the low and high SES neighborhoods in NMX. We used 16S rRNA amplicon sequencing to characterize the house dust, which generally supports taxonomic resolution only at approximately the genus level, although species level differences may affect human health. Shallow or deep shotgun metagenomics, although more expensive, would improve microbial identification, and deep shotgun metagenomics would provide more accurate functional profiles of the samples. PICRUSt uses 16S rRNA data to extrapolate metagenome composition and provides relatively lower confidence functional pathway profiles of samples. Given that asthma is a common reason for presentation to outpatient clinics and household recruitment was through outpatient clinics, there may have been selection bias because families that had an asthmatic child in the home may have been more likely to enroll in the study. However, this bias could have been represented on both sides of the Border. We did not investigate differences in pollutants in the collected dust, such as metals or pesticides, which could affect the dust composition and microbial diversity, along with the growth of opportunistic bacterial pathogens^63^. The structural differences and occupant behavioral differences between US and MX led to the differences in microbial diversity in the household dust. However, many of these structural factors are correlated with each other, and larger sample sizes would be required to disentangle this. Although there were difference in microbial dust composition between homes with and without an occupant that reported having asthma this study did not account for how long those individuals with asthma had lived in that home.

In conclusion, despite TUS, NUS, and NMX being geographically close (< 100 km) and having similar climates, homes across this Border region differ in ways that lead to significantly different indoor environments. Mexican and US households differed in years of education; household income; the percentage of homes that had paved roads, flushing toilets, and piped water; the number rooms and bathrooms present in the home; and presence and type of cooling and flooring. Some of these household differences may have led to the significant differences we observed in the microbial composition of the house dust collected from MX or US homes. The dust from the from Mexican homes was enriched with *A. Alishewanella Paracoccus*, *Rheinheimera* genera and *Intrasporangiaceae family*. Future research should assess whether exposure to these bacteria during critical windows in early life may offer protection from development of asthma or allergic disease.

## Methods

### Study population

Patients at each of three clinics were approached from September to December of 2016 to participate in this study: El Rio Community Health Center in TUS (n = 20), Mariposa Community Health Center in NUS (n = 20), and the Secretaría de Salud de Sonora in NMX (n = 40). In NMX, 20 households were recruited from a traditionally high SES neighborhood (high SES) and 20 from a traditionally low SES neighborhood (low SES). All of the households that were recruited from the high SES neighborhood in NMX were in a subdivision neighborhood where the houses were constructed with identical layouts. Families were eligible to participate in this study if at least one parent was of Mexican descent and had at least one child younger than 5 years old. The University of Arizona Human Subjects Protection Program approved all study materials (IRB approval number: 1607687201), in addition to all necessary permissions and reviews from the US/Mexico Border Commission, the Secretaría de Salud de Sonora, Mariposa Community Health Center and El Rio Community Health Center.

### Questionnaire

During the home visit, a questionnaire was administered orally in English or Spanish by a trained research assistant to obtain information on household demographics, asthma prevalence, sanitation measures, drinking water sources, and pets in the home. Multiple household characteristics were assessed by the research assistant at the same time as the home visit(e.g., mildew, water damage, structural characteristics, and type and number of bathrooms).

### Dust sample collection

House dust was collected using a Hoover CH3000 vacuum cleaner equipped with a pre-weighed sterilized X-Cell 100 dust collection sock (Midwestern Filtration, Cincinnati, OH) inserted in the crevice tool. To collect floor dust, a one-meter square template was laid on the floor in the child’s room and vacuumed for five minutes. If a child did not have their own room, then the sample was collected in the room where the child regularly spends most of their time. The sock filter holding the collected dust was placed in a plastic bag sterilized under UV light in a hood. The vacuum and its accessories were cleaned with disinfectant wipes and sprayed with isopropyl alcohol between sampling each house. Collected dust samples were transported in a cooler with ice packs to the laboratory in the Medical Research Building at the University of Arizona in Tucson, AZ.

In the laboratory, each dust sample was transferred from the filter sock to a pre-weighted 50 ml sterilized centrifuged tube. The centrifuge tube with the dust sample was weighed three times using a Mettler Toledo AB54 Precision Balance Weight Scale (Mettler Toledo International, Inc., Columbus, OH). The average of three measurements was then recorded. All environmental samples were stored in a − 80 °C freezer until analyzed. Frozen dust samples were shipped to the Pathogen and Microbiome Institute at Northern Arizona University for amplicon library preparation and sequencing.

### DNA extraction

DNA was extracted using the MoBio Powersoil DNA isolation kit (Qiagen) with an additional mechanical lysis. Briefly, samples were placed in a lysing matrix E tube (MP Biomedical) with 600 µl of Buffer RLT Plus and lysed in 30 s increments for a total of 6 min at 10,000 × g. Samples were sat resting for 30 s between each bead beating to prevent heating. Extraction continued following the manufacturer's protocol. DNA was quantified using a NanoDrop 2000. Extraction blanks, which did not contain any sample during the extraction, were carried throughout the entire extraction and 16S rRNA gene sequencing.

### 16S rRNA gene sequencing

Sample processing and sequencing were performed using the Earth Microbiome Project (www.earthmicrobiome.org) protocols. The barcoded primers 515F/806R were used to target the V4 region of the 16S rRNA gene, as previously described^64^. Each PCR reaction contained 2.5 µl of PCR buffer (TaKaRa, 10 × concentration, 1 × final), 1 µl of the Golay barcode tagged forward primer (10 µM concentration, 0.4 µM final), 1 µl of bovine serum albumin (ThermoFisher, 20 mg/mL concentration, 0.56 mg/µl final), 2 µl of dNTP mix (TaKaRa, 2.5 mM concentration, 200 µM final), 0.125 µl of HotStart ExTaq (TaKaRa, 5 U/µl, 0.625 U/µl final), 1 µL reverse primer (10 µM concentration, 0.4 µM final). All PCR reactions were filled to a total 25 µL with PCR grade water (Sigma-Aldrich) then placed on a ThermalCycler. ThermalCycler conditions were as follows: 98 °C denaturing step for 2 min, 30 cycles of 98 °C for 20 s, 50 °C for 30 s, 72 °C for 45 s, and a final step of 72 °C for 10 min. PCR was performed in triplicate for each sample and an additional negative control was included for each barcoded primer. A post-PCR quality control step was performed using a 2% agarose gel (ThermoFisher). Extraction blank controls were processed through the 16S PCR with the same methods as samples. Barcode primer NTCs controls were carried through the agarose gel step. If amplification was present for negative controls, the PCR was repeated with a new barcoded 806R primer. Following agarose gel, PCR product was quantified using the Qubit dsDNA High Sensitivity Kit (ThermoFisher) and the Qubit fluorometer 4. PCR products were pooled at equimolar concentrations of 50 ng. Quality of the pool was assessed with the Bioanalyzer DNA 1000 chip (Agilent Technologies), combined with 1% PhiX and sequenced on the Illumina MiSeq using the 600-cycle MiSeq Reagent Kit VX (Illumina).

### Data analysis

#### Demographic and housing characteristic analysis

The house and family characteristics were analyzed by country (US, MX) and by neighborhood (TUS, NUS, high SES NMX, low SES NMX). Data analysis was conducted with Stata v16. (StataCorp, College Station, TX). Comparisons between the two countries were assessed with Fisher’s Exact and Mann Whitney U tests. Comparisons between the neighborhoods were made using Fisher’s Exact and Kruskal–Wallis tests. Non-parametric tests were used, as the data had a skewed distribution as tested by the Shapiro–Wilk test. An alpha level of 0.05 was considered statistically significant.

#### Microbial data analysis

The microbiome sequencing data was analyzed using QIIME 2 2021.2^65^. The emp-paired (Hamady & Knight 2009) action in the q2-demux plugin was used to demultiplex the data. We used the denoise-paired action in the q2-dada2^66^ plugin to perform sequence quality control and define amplicon sequence variants (ASVs) with the following parameter settings: trim-left-f = 0; trim-left-r = 0; trunc-len-f = 200; trunc-len-r = 230. Replicate samples were grouped together using median ceiling to average abundances. A phylogenetic tree was created using align-to-tree-mafft-fasttree^67,68^ in q2-phylogeny, for use with phylogenetic alpha and beta diversity metrics. q2-diversity’s core-metrics-phylogenetic action was used to compute Faith’s Phylogenetic Diversity Index^69^, Unweighted Unifrac^70^ and Weighted Unifrac^71^ at an even sampling depth of 24,090. The alpha-rarefaction^72^ action in the q2-diversity plugin was used to generate rarefaction curves based on Faith’s Phylogenetic Diversity Index^69^ to confirm that the richness of the samples was stable around the chosen sampling depth. The beta-group-significance action in the q2-diversity plugin was used to run PERMANOVA pseudo-F tests on across sample groupings of interest. False-discovery-rate (FDR) correction was applied to correct for multiple comparisons. These FDR-corrected *p*-values will be presented as “q-values” in this text. Taxonomic annotation of ASVs was performed using the qiime feature-classifier^73,74^ classify-sklearn method using the SILVA 138 classifier^75^. A taxonomic bar plot was generated for the data based on the SILVA-based taxonomy. An ANCOM analysis was applied to identify differentially abundant taxa at the genus level across cities and countries using qiime composition ancom^76^. Finally, a random forest model was built using the qiime sample-classifier^73,74^ classify-samples method to predict the country and neighborhood of origin of the microbiome sample. This model was trained on the ASV table combined with the phylum and genus tables that were generated by collapsing ASV into taxa using the collapse action in the q2-taxa plugin. The classifier used fivefold cross-validation, where 80% of the microbial data was used for training and the remaining 20% was used for testing in 5 iterations such that all samples are used both in training and testing, and the overall performance is averaged across the iterations. To predict the abundance of gene families and related functional pathways of microbial communities present in the house dust, a Phylogenetic Investigation of Communities by Reconstruction of Unobserved States (PICRUSt) analysis was completed, which is used to predict metabolic pathways based on 16S rRNA results. The significant differences between the functional pathways present in the US vs MX or the different neighborhoods’ dust was analyzed using MaSaLin2 software^77^. TUS was used as reference when comparing the functional pathways in the dust between the communities (i.e., high SES NMX, low SES NMX and NUS).

### Ethics approval

This study was performed in line with the principles of the Declaration of Helsinki. Approval was granted by The University of Arizona Human Subjects Protection Program (IRB approval number: 1607687201) in addition to all necessary permissions and reviews from the US/Mexico Border Commission, the Secretaría de Salud de Sonora, Mariposa Community Health Center and El Rio Community Health Center.

### Consent to participate

Informed consent was obtained from all individual participants included in the study.

### Supplementary Information

Supplementary Information.

## Data Availability

All 16S rRNA and deidentified sample metadata has been submitted to the SRA under bioproject ID PRJNA1018913. The data will be made publicly accessible through this SRA submission upon publication.
