# Supplementary material for: Environmental and structural factors associated with bacterial diversity in household dust across the Arizona-Sonora border
Source: Sci Rep. 2024 Jun 4;14:12803. doi: 10.1038/s41598-024-63356-6 (PMC11150412; doi:10.1038/s41598-024-63356-6)
Supplement: Supplementary file 1 — Supplementary Information. [file 41598_2024_63356_MOESM1_ESM.docx]

**Figure S1. Dust floor load (mg/m^2^) by country and flooring type given that only 1 home had dirt floors this dust floor loading for that home is excluded** Abbreviations**:** MX=Mexico; US= United States of America

**
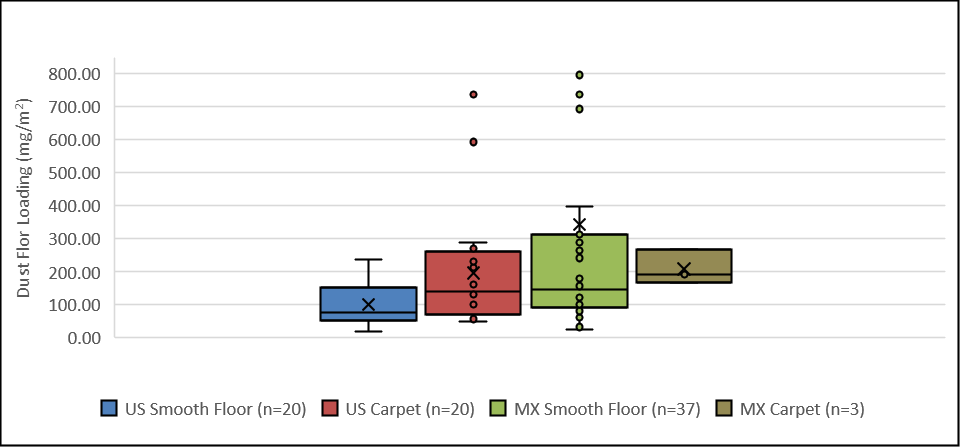
**

**Figure S2. : A**. Unweighted UniFrac beta diversity plot comparing households with central air condition, evaporative colling, or nothing, (p=0.006 comparing central air conditioning vs nothing p=0.069 having both central air conditioning and evaporative cooling vs nothing, p=0.01 having Evaporative cooling vs nothing). **B:** Unweighted UniFrac beta diversity plot comparing households on asphalt vs unpaved or dirt roads (p=0.01) **C.** Unweighted UniFrac beta diversity plot comparing households that answered the questionnaire saying they had an asthmatic in the household vs they did not have an asthmatic in the household (p=0.18)


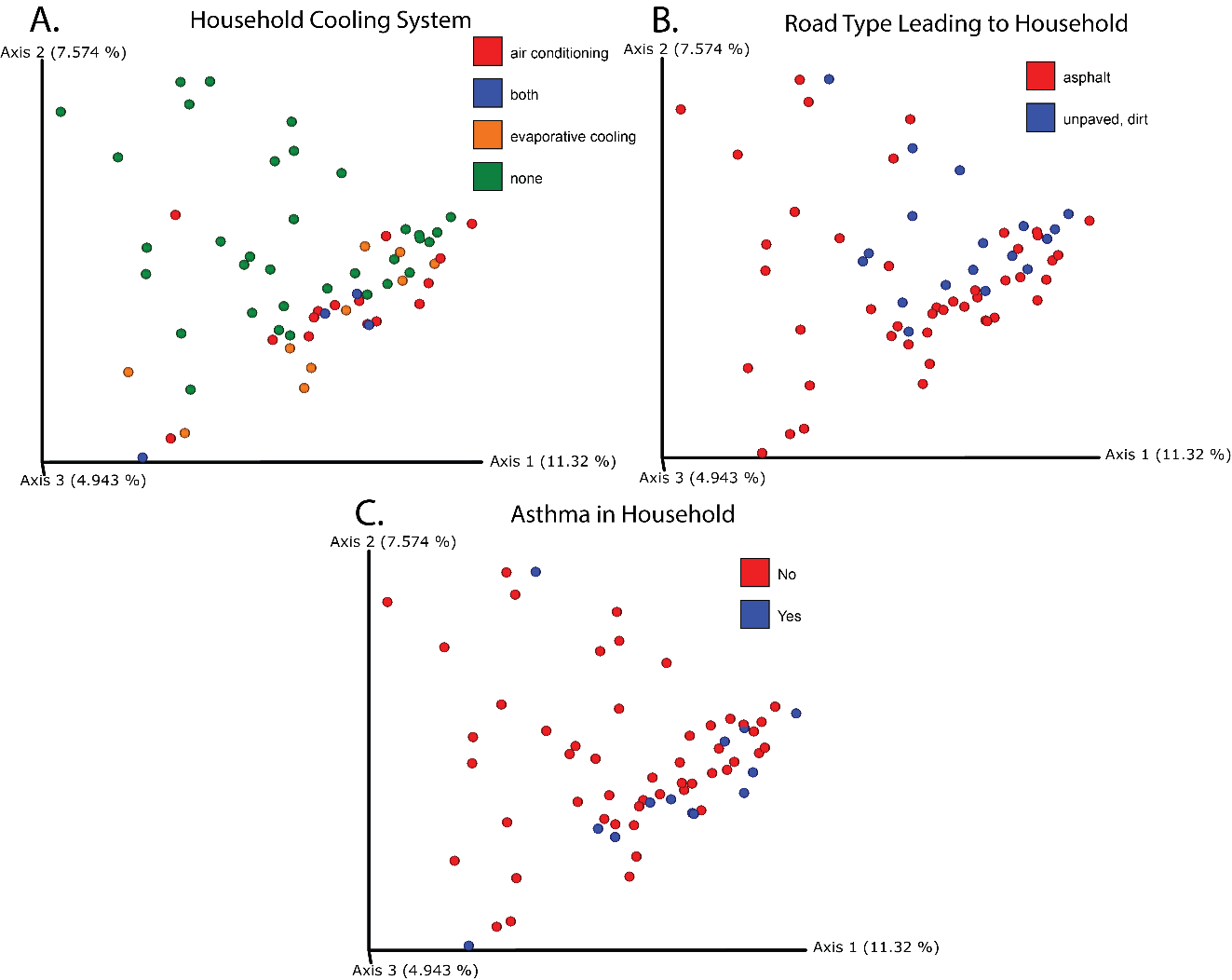


**Figure S3.** ANCOM and Sample classifier comparing the house dust from homes in the US and MX by city and neighborhood. HSES is the high socioeconomic neighborhood in Nogales, MX and LSES is the low socioeconomic neighborhood in Nogales, MX. Low SES NMX= low socioeconomic status Nogales, Sanora, Mexico; High SES NMX= high socioeconomic status Nogales, Sanora, Mexico; MX=Mexico; NUS=Nogales, Arizona, United States of America; TUS= Tucson, Arizona, United States of America; US= United States of America.  The x-axes in these figures represent the relative abundance of the taxon that is highlighted in the panel, while the y-axes represent the categorizations of the samples. Each point therefore represents the relative abundance of a taxon in a single sample, and the box plots and the histograms show the distribution of the relative abundances in each sample group.


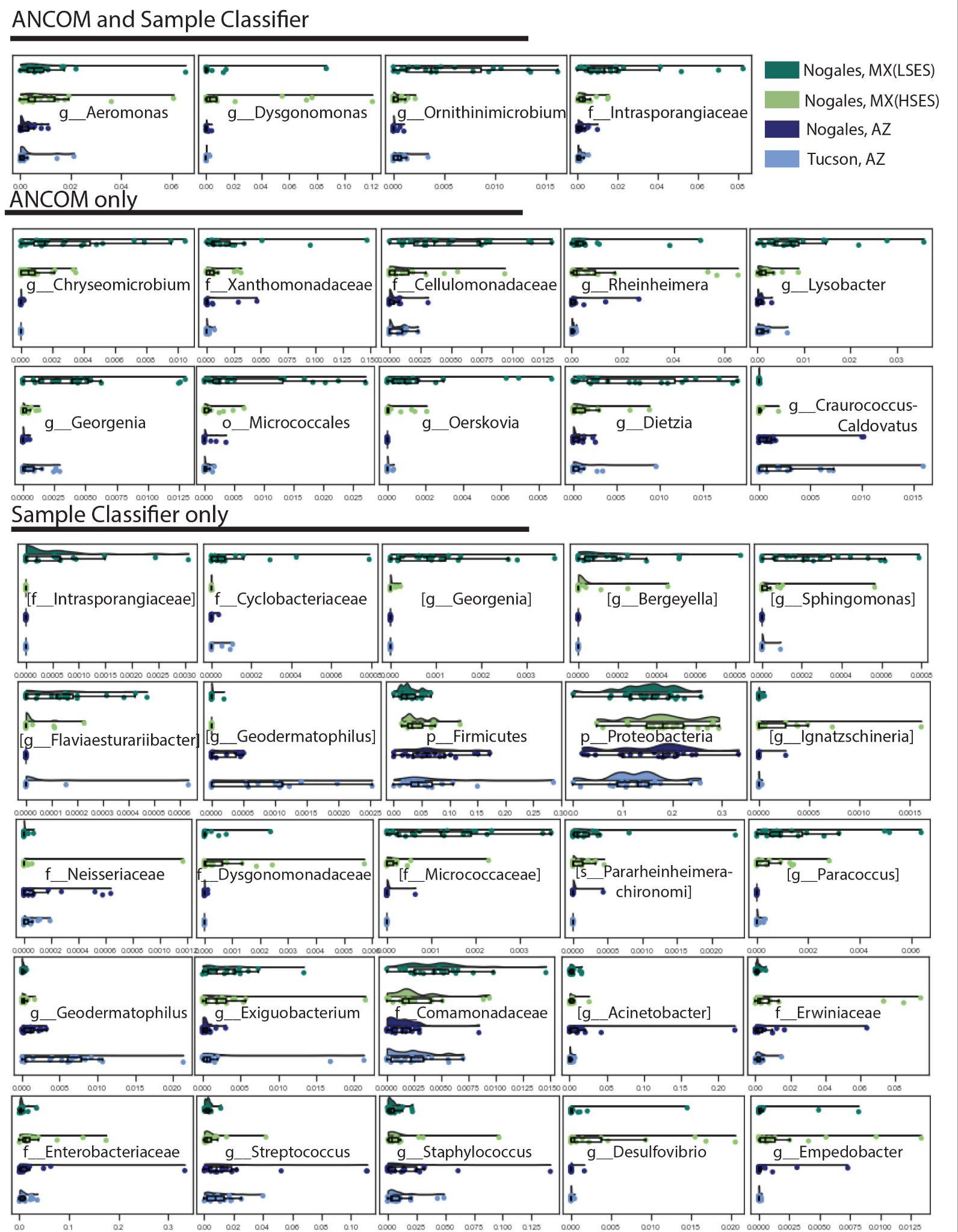


**Figure S4**: Microbial functional pathways found to be significantly different between the US and MX house dust. Phylogenetic Investigation of Communities by Reconstruction of Unobserved States (PICRUSt) analysis was used to extrapolate function pathways from 16S rRNA data. MaSaLin2 software was used to compare the functional pathways for significance. Abbreviations: Low SES NMX= low socioeconomic status Nogales, Sanora, Mexico; High SES NMX= high socioeconomic status Nogales, Sanora, Mexico; MX=Mexico; NUS=Nogales, Arizona, United States of America; TUS= Tucson, Arizona, United States of America; US= United States of America


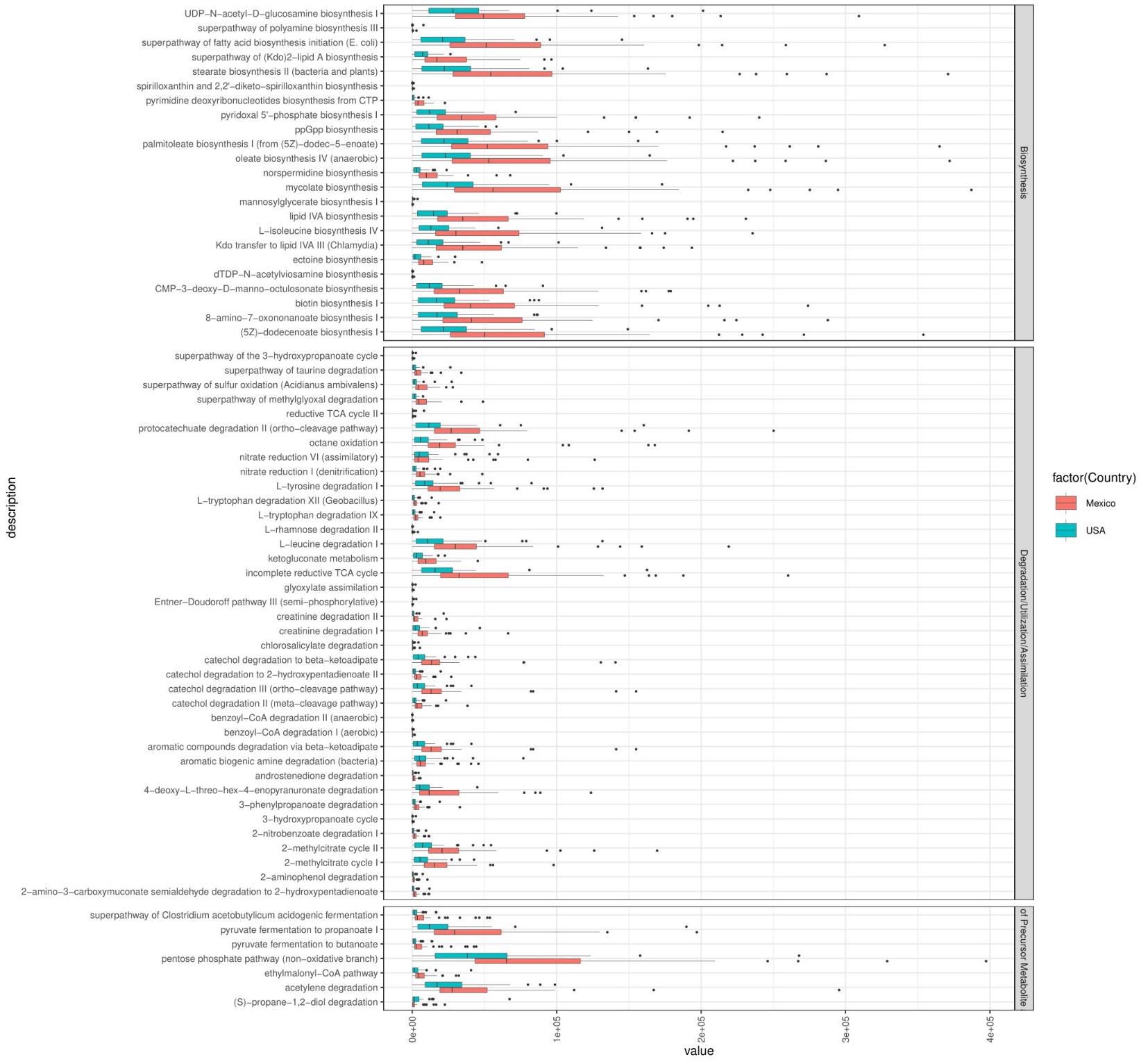


**Figure S5**. Heat map of microbial functional pathways found to be significantly different between the high SES neighborhood in NMX vs the low SES NMX, NUS vs TUS with TUS used as the reference. Phylogenetic Investigation of Communities by Reconstruction of Unobserved States (PICRUSt) analysis was used to extrapolate function pathways from 16S rRNA data. MaSaLin2 software was used to compare the functional pathways for significance. Blue represents pathway depletion whereas red represents pathway enrichment. Abbreviations: Low SES NMX= low socioeconomic status Nogales, Sanora, Mexico; High SES NMX= high socioeconomic status Nogales, Sanora, Mexico; MX=Mexico; NUS=Nogales, Arizona, United States of America; TUS= Tucson, Arizona, United States of America; US= United States of America


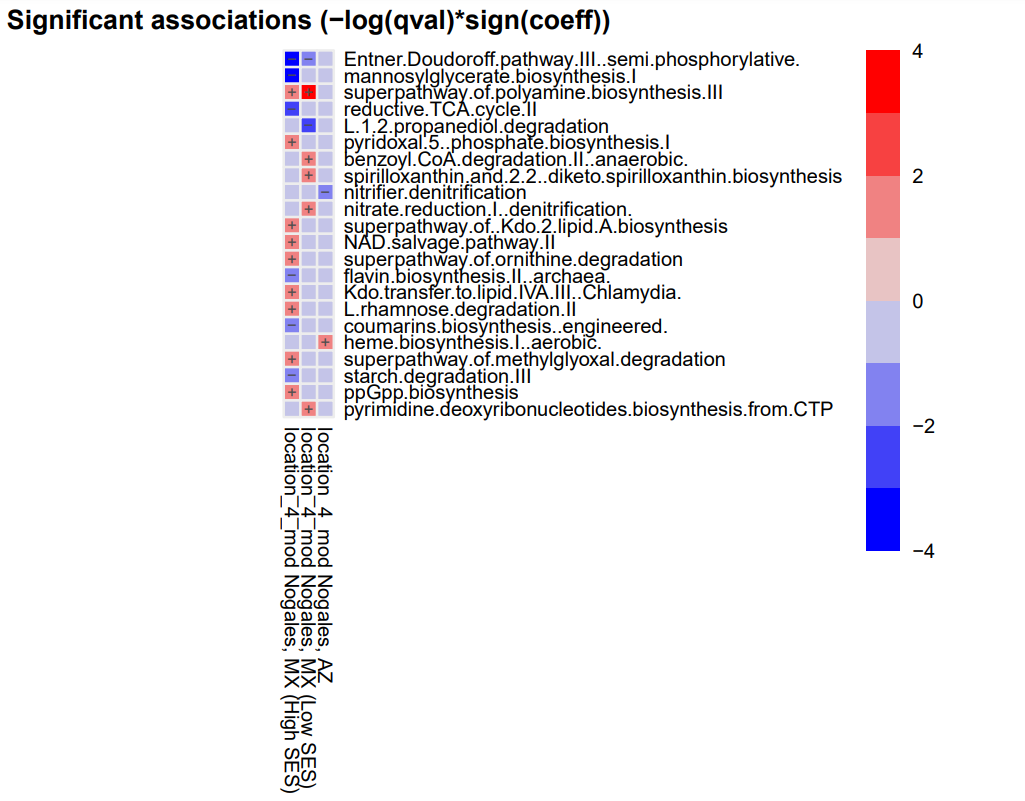


Table S1: Unweighted UniFrac beta diversity values and difference in household dust comparing houses based on physical characteristics and occupant characteristics of homes analyzed. Beta biodiversity were calculated in each category comparing one value or household characteristic to the other within that category.

| Variable | Categories | Sample size | Pseudo-F | p-value | q-value |
| --- | --- | --- | --- | --- | --- |
| Frequency of floor cleaning | Every day (Ref) |  |  |  |  |
|  | <1 time/month | 26 | 1.28 | 0.10 | 0.45 |
|  | 1 time/week | 25 | 0.93 | 0.63 | 0.78 |
|  | Several times/day | 35 | 1.14 | 0.22 | 0.54 |
|  | Several time/week | 39 | 1.51 | 0.005 | 0.05 |
|  | <1 time/month (ref) |  |  |  |  |
|  | 1 time/week | 3 | 0.83 | 1.00 | 1.00 |
|  | Several times/day | 13 | 1.05 | 0.30 | 0.58 |
|  | Several time/week | 17 | 0.98 | 0.40 | 0.58 |
|  | 1 time/week (ref) |  |  |  |  |
|  | Several times/day | 12 | 0.80 | 0.92 | 1.00 |
|  | Several time/week | 16 | 0.96 | 0.39 | 0.58 |
|  | Several times/day (ref) |  |  |  |  |
|  | Several time/week | 26 | 1.25 | 0.14 | 0.45 |
| Type of flooring | All rug/carpet (ref) |  |  |  |  |
|  | All smooth floor | 49 | 1.79 | 0.01 | 0.03 |
|  | Both-mixture | 13 | 0.88 | 0.79 | 0.79 |
|  | All smooth floor (ref) |  |  |  |  |
|  | Both-mixture | 44 | 1.02 | 0.38 | 0.56 |
| Type of housing structure | Detached house (ref) |  |  |  |  |
|  | Duplex/attached house | 48 | 1.25 | 0.12 | 0.30 |
|  | Multi-unit (apartment) | 40 | 1.04 | 0.31 | 0.31 |
|  | Trailer/mobile | 37 | 1.38 | 0.14 | 0.30 |
|  | Duplex/attached house (ref) |  |  |  |  |
|  | Multi-unit (apartment) | 16 | 1.18 | 0.15 | 0.30 |
|  | Trailer/mobile | 13 | 1.12 | 0.30 | 0.31 |
|  | Multi-unit (apartment) (ref) |  |  |  |  |
|  | Trailer/mobile | 5 | 1.53 | 0.22 | 0.31 |
| Household Income | $16,000-$20,999 (ref) |  |  |  |  |
|  | $21,000-$25,999 | 10 | 0.94 | 0.52 | 0.64 |
|  | <$6,000 | 35 | 1.50 | 0.02 | 0.17 |
|  | ≥$26,000 | 12 | 1.21 | 0.07 | 0.21 |
|  | $21,000-$25,999 (ref) |  |  |  |  |
|  | <$6,000 | 33 | 1.55 | 0.03 | 0.17 |
|  | ≥$26,000 | 10 | 1.10 | 0.23 | 0.42 |
|  | $6,000-$9,999 (ref) |  |  |  |  |
|  | $16,000-$20,999 | 8 | 1.20 | 0.15 | 0.37 |
|  | $21,000-$25,999 | 6 | 1.06 | 0.31 | 0.49 |
|  | <$6,000 | 31 | 0.88 | 0.69 | 0.74 |
|  | ≥$26,000 | 8 | 1.13 | 0.33 | 0.49 |
|  | $10,000-$15,999 (ref) |  |  |  |  |
|  | $16,000-$20,999 | 13 | 0.85 | 0.75 | 0.75 |
|  | $21,000-$25,999 | 11 | 0.94 | 0.45 | 0.62 |
|  | $6,000-$9,999 | 9 | 1.08 | 0.21 | 0.42 |
|  | <$6,000 | 36 | 1.48 | 0.05 | 0.18 |
|  | ≥$26,000 | 13 | 0.90 | 0.69 | 0.74 |
|  | <$6,000 (ref) |  |  |  |  |
|  | ≥$26,000 | 35 | 1.87 | 0.003 | 0.045 |
| Mildew presence | No Mildew (ref) |  |  |  |  |
|  | Yes mildew | 52 | 1.13 | 0.20 | 0.20 |
| Moisture presence | No moisture (ref) |  |  |  |  |
|  | Yes moisture | 54 | 1.07 | 0.28 | 0.28 |
| Piped water | No piped water (ref) |  |  |  |  |
|  | Yes piped water | 54 | 1.75 | 0.008 | 0.008 |
| Flushing Toilets | No flushing toilets (ref) |  |  |  |  |
|  | Yes flushing toiles | 54 | 1.52 | 0.03 | 0.03 |
| Smokers present in the home | No smokers (ref) |  |  |  |  |
|  | Yes smokers | 54 | 0.85 | 0.80 | 0.80 |
| Primary Drinking water Source | Bottled water (ref) |  |  |  |  |
|  | Hauled in water | 36 | 1.47 | 0.04 | 0.08 |
|  | Indoor, public water | 32 | 1.22 | 0.12 | 0.14 |
|  | Vending machine water | 29 | 1.27 | 0.08 | 0.12 |
|  | Hauled in water (ref) |  |  |  |  |
|  | Indoor, public water | 22 | 1.56 | 0.005 | 0.015 |
|  | Vending machine water | 19 | 1.62 | 0.005 | 0.0150 |
|  | Indoor, public |  |  |  |  |
|  | Vending machine water | 15 | 0.97 | 0.50 | 0.50 |
